# Understanding Moral Distress among Eldercare Workers: A Scoping Review

**DOI:** 10.3390/ijerph19159303

**Published:** 2022-07-29

**Authors:** Risto Nikunlaakso, Kirsikka Selander, Elina Weiste, Eveliina Korkiakangas, Maria Paavolainen, Tiina Koivisto, Jaana Laitinen

**Affiliations:** Finnish Institute of Occupational Health, P.O. Box 40, FI-00032 Työterveyslaitos, Finland; kirsikka.selander@ttl.fi (K.S.); elina.weiste@ttl.fi (E.W.); eveliina.korkiakangas@ttl.fi (E.K.); maria.paavolainen@ttl.fi (M.P.); tiina.koivisto@ttl.fi (T.K.); jaana.laitinen@ttl.fi (J.L.)

**Keywords:** moral distress, eldercare, scoping review, interventions

## Abstract

The aging of the population in Western countries will increase the use of social and health services in the future. Employees in eldercare are at risk for experiencing moral distress, which is associated with poor work ability. The causes and consequences of moral distress among eldercare workers remain undiscovered. This scoping review investigates the existing studies of causes and consequences of moral distress among eldercare workers. Additionally, it seeks evidence of interventions designed to mitigate moral distress in eldercare workers. Fourteen studies were included in the final review. Most of the included studies were qualitative, aiming to increase understanding of morally challenging situations in eldercare. We also found quantitative studies with cross-sectional designs and small sample sizes. Thus, no reliable evidence of causal effects between moral distress and worker wellbeing in eldercare was found. We found no interventions undertaken to resolve moral distress among eldercare workers, either. More research is needed on the causes and consequences of moral distress and on interventions to mitigate moral distress among eldercare workers. This is of utmost importance to increase the attractiveness of eldercare as a workplace and to improve eldercare workers’ ability to work and sustain long working careers.

## 1. Introduction

The aging of the population in Western countries will increase the use of social and health services (HSS) in the future. HSS employees are retiring rapidly; at the same time, young age groups entering the HSS workforce are significantly smaller. A higher number of elderly people and a diminishing workforce pose major challenges to HSS in Western countries. Lack of workforce and simultaneous work stressors, such as moral distress, high job strain, effort–reward imbalance, and organizational injustice, increase the risk of poor work ability, especially when accumulating on the same individuals [1].

HSS employees regularly encounter ethical dilemmas when ethical traditions and principles in care, such as patient autonomy and beneficence, conflict [2]. This can lead to moral distress [2], which is associated with poor work ability [1]. Earlier studies have described ethical dilemmas in public health care [3,4], in clinical nursing [2,5], and among nursing managers [6,7]. The dilemmas are caused by patient-level determinants (e.g., balancing integrity and patient protection, and conflicts and mistrust with patients and their families), unit/team level determinants (e.g., conflicts with colleagues and the management system, and poor organization of the working process), and system-level determinants (e.g., limits of care, and employee shortage) [2,3,8,9,10]. Consequently, the employees are found to experience negative emotions such as frustration and anger, emotional exhaustion, cynical attitudes, and depersonalization toward patients, resulting in quitting or considering quitting their position [10]. Ethical dilemmas and moral distress should be distinguished from more acute and traumatizing care-related effects, such as second victim effects and moral injuries, which result from unforeseeable and acute incidents during treatment [11,12].

The employees in eldercare face work-related stress (high workload, high responsibilities, reduction in rest periods, contributory inequity, high turnover), and are at particular risk of experiencing moral distress at their work [11,13]. Elderly people often have many severe diseases and need help with everyday life activities. Thus, eldercare workers often are exposed to strong emotional experiences related to elderly people and ethically challenging situations.

A few studies on moral distress in the eldercare setting have been conducted. A recent systematic review [14] identified research focusing on ethical and legal issues in geriatric care and overviewed existing grievances and possible solutions to take care of old patients in both an ethical and legally correct way. It showed that the main ethical dilemmas in eldercare are caused by patient-level determinants, such as patients’ autonomy; respect for their needs, wishes and values; and respect for their decision-making [14]. Prior research has also noted that in eldercare, ethical dilemmas are caused by unit/team-level determinants, such as staff conflicts [15] and limited time for providing care [16], as well as system-level determinants such as lack of financial resources and organizational problems [17].

Despite a recent review of ethical issues in eldercare [14], a gap in the knowledge of causes and consequences of moral distress among workers in eldercare remains. Additionally, more evidence is needed to develop interventions that are designed to mitigate moral distress specifically in eldercare. Understanding the causes and consequences of moral distress in eldercare can help nursing managers and policy makers design strategies to cope with them. New strategies are needed to increase the attractiveness of eldercare and to improve eldercare workers’ work ability.

## 2. Aim of the Study

This scoping review aims to investigate the causes and consequences of moral distress in eldercare. In addition, it aims to seek evidence of interventions designed to mitigate moral distress in this specific sector.

The research questions are:

(1) What factors cause moral distress in eldercare?
(2) What are the consequences of moral distress for employees’ wellbeing?
(3) What kind of interventions to mitigate moral distress have been implemented in eldercare?

Following the rationale of Fourie [18], we define and use moral distress in a broad sense, as the psychological response to morally challenging situations such as those of moral constraint or moral conflict, or both.

## 3. Methods

We conducted a scoping review following the PRISMA-ScR protocol for scoping reviews [19]. A systematic literature search for relevant studies was conducted in February 2022 in the PubMed, Scopus, and CINAHL databases, which we considered the most relevant for moral distress and studies in the eldercare domain. Although our focus was on moral distress, we used multiple keywords and titles to avoid missing studies which may have used different terminology (see Appendix A). A librarian from the University of Eastern Finland helped with the search strategy.

Studies were selected based on the following eligibility criteria:

1. We included studies where the target population comprised employees working in eldercare services and in close contact with elderlies. We excluded studies of services with multiple patient groups, such as palliative care, where elderlies are one among multiple patient groups. We also excluded studies of eldercare managers who had little contact with elderlies.
2. We included studies which focused on eldercare workers’ moral distress. We excluded studies which examined moral distress in caregiving in general or examined it from the clients’ perspective.
3. We included studies published in 1997–2022, written in English and published in peer-reviewed journals.
4. We included both qualitative and quantitative studies, excluding validation studies and case studies.

**Figure 1:**
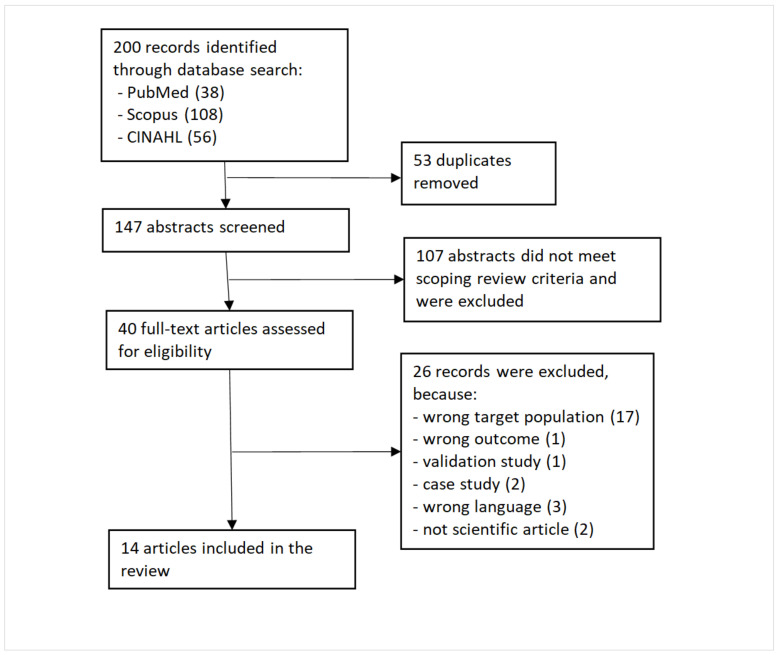
Flow chart.

We identified 200 studies in database searches, of which 53 were duplicates (see Figure 1). Abstract screening revealed 107 irrelevant studies based on eligibility criteria, resulting in 40 full-text studies, which were assessed for eligibility. Of these, 26 were excluded, mainly due to wrong population (17 studies), wrong language (3), other than original journal article (2), wrong method (1 validation study and 1 case study), and wrong outcome (1). The remaining 14 studies were included in the review. We used Rayyan software to assist with the screening process. Abstract screening was conducted separately by two readers (RN, KS) whereafter conflicts were discussed and resolved. In the full-text screening, 40 studies were distributed among five researchers (RN, KS, EK, EW, and MP), who analyzed them in pairs. Disagreements were resolved by the whole writer group.

Five researchers (RN, KS, EK, EW, and MP) independently collected relevant information on the remaining 14 whole-text articles on a Microsoft Excel worksheet. We collected information on the publication year, the country where the study was conducted, the study design, the target population, the study size (n), the definition of moral distress, how moral distress was measured, what causes or affects moral distress, and the effects of moral distress. Thereafter, two authors (RN and KS) formed a synthesis of the Excel worksheet.

## 4. Results

Fourteen studies were included in the final review (see Table 1). Most were published in 2019 (3) or 2020 (3). Ten articles were published after 2015, which indicates a growing interest in moral distress in eldercare. Most studies were conducted in Europe (7: France (1), Sweden (1), Norway (1), UK/England (2), Belgium (1), Italy (1)) or Canada (4). Two studies were conducted in Asia (Iran and Korea) and one in the USA. Most studies were qualitative interviews (7), four studies used a quantitative study design, and three combined both qualitative and quantitative approaches. All the quantitative studies were based on rather small sample sizes, ranging between 154 and 389 observations. Furthermore, none of the quantitative studies had a longitudinal design. No intervention studies were found.

### 4.1. Target Populations in Studies

In most of the included studies (8), the target population comprised several eldercare professions, such as nurses, practical nurses, care/nurse assistants, physicians, psychologists, physiotherapists, and case managers. Four studies concentrated on nurses, one on physicians, and one on various professionals that were responsible for digital service transformation (e.g., head of unit, digitalization strategist, quality assurance developer, or chief nurse).

### 4.2. Definitions of Moral Distress

Definitions varied widely in the included studies. Two studies adapted Jameton’s [20] definition of moral distress, which is widely used in the moral distress literature. Two studies used the definition from Nathaniel’s [21] dissertation work. In three studies, the researchers formed their own definition based on previous literature. One study had no definition at all. Five studies had their own unique citation for the definition of moral distress (see Table 1), and one had its own definition without a citation.

Since the included studies were conducted in different countries, they might have discussed cultural variety in moral distress definitions. Corvol et al. [22] found differences in ethical values and in experiencing ethical issues between French and American contexts, and Sedaghati et al. [23] found moral distress levels to differ in various regions of Iran. Other studies had no discussion of cultural variations.

### 4.3. How Moral Distress Was Measured

Most of the included studies used a qualitative design with open-ended questions and some examples of themes or questions (8). Two articles had no examples of themes or questions to guide interviews.

In quantitative studies, three research groups developed their own moral distress scale and three adapted a scale used before. The adapted scales were a scale by Preshaw [24], a modified version of Corley’s [25] moral distress scale, and Wocial and Weaver’s [26] moral distress thermometer.

### 4.4. What Causes or Affects Moral Distress

We identified causes and consequences of moral distress in the included studies, finding a total of 53 causes and 21 consequences (Table 2 and Table 3). We classified them into data-based thematic categories: the causes into 4 main categories and 9 subcategories and the consequences into 4 main categories.

The first subcategory, Organizational policies and leadership of the category Organizational restraints, consisted of causes stemming from the particular organization the eldercare workers work in: leadership, rotation systems, and policies regarding who are treated and how. It comprised 13 causes identified in 9 included articles. The second subcategory, Inadequate resources and resource allocation, also included causes stemming from organizational constraints, but it focused directly on the work resources and their allocation. It comprised 8 causes identified in 7 included articles.

The category Relational and power-related issues included problems stemming from the hierarchical nature of the organization, conflicts with or unaccountability of colleagues, and challenging relationships with patients and their families. The subcategory Hierarchy issues comprised 3 causes in 3 included articles; Relations with colleagues, 5 causes in 3 articles; and Relations with patients and families, 8 causes in 8 articles.

Our third category was Moral distress in caring for patients. This included inadequate or futile care of patients (4 causes in 2 articles), seeing patients suffering (4 causes in 3 articles) and conflicts with the values of autonomy and beneficence (3 causes in 2 articles).

Our fourth category included causes stemming from eldercare workers’ assessment of their own competence and feeling of safety when caring for challenging patients. It comprised 5 causes in 5 articles.

**Table 2:** Causes of moral issues and their categorizations identified in the included studies. Each study is indicated using study numbers in Table 1.

Causes of Moral Distress: Main Category	Causes of Moral Distress: Sub-Category	Causes of Moral Distress	Number of Causes
Organizational restraints	Organizational policies and leadership (in 9 studies)	Administration quantifies the performance of the nurses (1), neglecting non-registered clients (1), no time with patients in the rotating system for visiting care (1), organizational interest in becoming digital vs. duty of care for the patients (3), lack of trust in the healthcare organization (4), conflict between what workers felt right and what was the duty of care (5), acceptance of work pressure (8), inconsistent care expectations (9), leadership failures (10), inadequate care models and resources (10), a culture of tasks over touch (10), policies not in the best interest of the patients (11), fearing consequences if advocating for residents (14),	13
	Inadequate resources and resource allocation (in 7 studies)	allocation of resources (2), structure and quality of services and resource allocation within the nursing home (5), lack of staff (7), having to rush the care and making patients wait (7), being forced to provide low-quality care to reduce costs (8), lack of resources (9), lack of time, balancing residents’ needs (11, 12)	8
Relational and power-related issues	Hierarchy/power to influence care (in 3 studies)	Lack of control over the development of digital solutions (3), relations between physicians and nurses (8), undervaluing expertise (11)	3
	Relations/conflicts with colleagues (in 3 studies)	Working with incompetent/unaccountable colleagues (6, 9, 10) not involved in end-of-life decisions (6), lack of ethical debate (6),	5
	Relationship between patients/families and professionals (in 8 studies)	Differences in the understanding of moral and legal responsibility of care (4), conflicts within the relationships of staff, families, and residents (5, 9, 14), families do not provide necessities such as clothing (7), conflicting expectations with families around care (10), difficult communication (12, 14)	8
Caring for patients	Inadequate/futile care (in 2 studies)	Unjustifiable life support (6), unnecessary tests and treatments (6), decision about life-sustaining treatment (13), discordance when preferring a more comfort-focused plan than the patient is receiving (13)	4
	Patients suffering (in 3 studies)	Working in acute (vs. chronic) geriatric care (6), seeing a low quality of life due to lack of activities (7), proximity to the bedside (7), unpredictable dying trajectories (14)	4
	Patient autonomy (in 2 studies)	Refusal of care (2), collecting and sharing personal patient data (2), balancing autonomy, care and dignity (4)	3
Workers’ self-assessment	Lack of competence & safety issues (in 5 studies)	Acknowledgement of visiting nurses’ limitations (1), unprepared to use digital solutions (3), managing patients’ dementia behaviors (9), remaining silent to avoid consequences (10), self-perceived weakness (12)	5

**Table 3:** Consequences of moral distress and their categorizations identified in the included studies. Each study is indicated using study numbers in Table 1.

Consequences of a Moral Distress: Main Category	Consequences of Moral Distress	Number of Consequences
1. Avoidance and resistance (in 4 studies)	Intentional or actual job leave (6), reduced job satisfaction and wanting to quit job (8), quitting job (10), sick leave (10), negotiating the organizational constraints in various, often creative ways (11)	5
2. Mental health problems (in 10 studies)	Distress and anxiety (1), distress (3, 5), exhaustion (4), burnout (7), psychological distress: feeling angry, powerless, and frustrated (8), feeling emotionally drained (8), emotional reactions (10), emotional distress (11, 12), helplessness and fear (12), powerlessness (14)	12
3. Unhealthy and asocial behavior (in 3 studies)	Engaging in unhealthy behaviors (8), relationship effects (9), distraction and isolation (12)	3
4. Physical symptoms (in 2 studies)	Physical exhaustion (8), physical reactions (9)	2

### 4.5. Consequences of Moral Distress on Workers

Most of the included studies examined both the causes and the consequences of moral distress on worker health and wellbeing. However, in Spenceley et al. [27] and Wocial et al. [26], moral distress was used as an outcome variable without further analysis of its consequences. In Corvol et al. [22], moral distress was defined as the consequence of conflicting values, and thus its consequences on worker wellbeing or health were not examined further.

Most of the studies also found moral distress to have a considerable impact on worker wellbeing. Sedaghati et al. [23] and Wocial et al. [26], instead, found the prevalence of moral distress low or moderate.

We found 4 studies with 5 consequences regarding workers’ avoidance and resistance of work, including job leave and sick leaves. Twelve mental health–related consequences were found in ten studies. These included distress, anxiety, and various emotional reactions. Unhealthy and asocial behavior was identified as a consequence 3 times in 3 articles. Finally, 2 studies found 2 consequences regarding physical reactions among eldercare workers.

### 4.6. Interventions to Mitigate Moral Distress

We found no studies of interventions to mitigate moral distress of eldercare workers. We did, however, find strategies which workers had tried or would want to try to mitigate moral distress (see Table 4) in three included studies. Two studies found four strategies which advocated organizational support and education. Two studies advocated utilizing peer support, and two studies advocated improving workers’ self-care and competence. Finally, one study suggested that healthcare workers, when facing conflicting values, often choose to defend the patient’s viewpoint.

## 5. Discussion

The current scoping review aimed to find the causes and consequences of moral distress among eldercare workers and to discover whether interventions to resolve moral distress exist. Most of the included studies were qualitative, aiming to increase understanding of the morally challenging situations in eldercare. We also found quantitative studies with cross-sectional designs and small sample sizes (from 154 to 389 respondents). Thus, no reliable evidence of causal effects between moral distress and worker wellbeing in eldercare was found. We found no interventions resolving moral distress among eldercare workers, either.

We did, however, find several factors associated with moral distress, which were reported as causes of moral distress in the included studies. After categorization of these causes, we found that organizational restraints were most often examined. Relational and power-related issues, issues with caring for patients, and self-assessed competence and safety issues were also examined. Most of the included studies also examined the consequences of moral distress. Work avoidance, mental health problems, unhealthy and asocial behavior, and physical symptoms were reported to result from moral distress. Although no interventions to resolve moral distress were found, three of the studies investigated strategies to mitigate moral distress. Organizational support and education, peer support, improving selfcare and competence, and defending patients were suggested in these studies.

Our findings of reported causes and consequences of moral distress are in line with, first, those of Rittenmeyer and Huffman [28], who, reviewing professional nurses’ moral distress, found institutional constraints, patients’ suffering and futile care, and unequal power hierarchies as major causes. They also found similar consequences of moral distress: biopsychosocial responses, powerlessness, and quitting the profession. Second, Podgorica et al. [14] found patient autonomy to be an important ethical issue in eldercare. Third, regarding relationships with families, Rainer et al. [2] found family conflicts to cause ethical conflicts among nurses. Our finding on eldercare workers’ self-assessed competence and safety issues seems to be a little-studied theme.

In addition to lacking reliable evidence of causal effects of moral distress, the included studies defined and measured moral distress in varying ways. Most of the studies reported examining associations of moral distress [23,27,29,30,31,32,33,34,35,36], while some analyzed ethical dilemmas [22,37,38,39]. Yet almost all the studies defined the concept of moral distress or ethical dilemma differently. Moreover, we found no consistency in how moral distress was measured. Each quantitative study used a different questionnaire, either self-developed or a reference to a validated instrument. Similarly, moral distress was discussed in the qualitative interviews using different questions.

We categorized the studies on the basis of our interpretation. Since there is no generally accepted nomenclature or classification for qualitative and quantitative studies, this could have introduced bias. However, we believe that our classification process, with major categories and subcategories, is transparent. As the current study is a scoping review, we did not perform a meta-analysis or a risk-of-bias assessment; moreover, both of these would have required a more in-depth data extraction and intervention or follow-up studies, so conducting them was not feasible. This review may also be subject to publication bias since we included only peer-reviewed, published studies written in English and ignored gray literature and other languages.

## 6. Conclusions

In future studies, more clarity on the definitions and measurements of moral distress is needed. Furthermore, more longitudinal and intervention studies with large samples are needed to obtain more information on the prevalence and causal inference of moral distress, worker health, and work ability in eldercare. Harmonization of methods would improve the comparability of the study results.

Since moral distress is an important job stressor, especially when clustering with other stressors [1], effective interventions should be developed. The strategies found in this scoping review can be used to plan future interventions.

This scoping review showed that more research is needed on the causes and consequences of moral distress and on interventions to mitigate moral distress among eldercare workers. With more knowledge, nurses and nursing students can be educated in handling morally challenging situations. This is of utmost important to increase the attractiveness of eldercare as a workplace and to improve eldercare workers’ ability to work and to sustain long working careers.

## Figures and Tables

**Table 1:** Characteristics of the included studies (N = 14) evaluating the causes and consequences of moral distress in eldercare.

Authors	Study Number	Title (Concept)	Country	Year	Study Mehodology	Target Population	Study Size/N	Definition of Moral Distress	How Moral Distress Was Measured
Choe et al.	1	Ethical concerns of visiting nurses caring for older people in the community (ethical dilemmas)	Korea	2015	Qualitative interviews	Nurses	13	No definition	Prior to the interviews, participants were asked to think of ethical concerns they might have experienced while working as visiting nurses. They were asked an open-ended question: ‘‘What ethical concerns have you ever experienced while working as a visiting nurse?
Corvol et al.	2	Ethical issues in the introduction of case management for elderly people (ethical dilemmas)	France	2013	Qualitative interviews and focus groups	Case managers with different background	12	Definition based on Beauchamp and Childress’s principles of biomedical ethics	Open-ended questions related to clients’ choice, cost control, information sharing, and other ethical issues that were not addressed by the interviewer.
Frennert	3	Moral distress and ethical decision-making of eldercare professionals involved in digital service transformation (moral distress)	Sweden	2020	Mixed-method: interview + survey	Various professionals responsible for municipal’s digital service transformation (e.g., head of unit, digitalization strategist, quality assurance developer, chief nurse care services coordinator, and business developer strategist)	interview 10; survey 40	Adapted and modified from Sidwick (2019)	In a survey, the first question was: “Do you encounter any ethical matters in regard to digital care services in your everyday work practices?” If they answered yes, exploratory open-ended questions followed concerning what kinds of ethical matters were faced, specifically which kinds of digital services, patients, and situations, and their influence on ethical decision-making.
Holm & Severinsson	4	Reflections on the ethical dilemmas involved in promoting self-management (ethical dilemmas)	Norway	2014	Qualitative focus group interviews	Various professionals	2 focus groups	Definition is based on several previous empirical works	No examples of questions in group sessions.
Muldrew et al.	5	Ethical issues experienced during palliative care provision in nursing homes (ethical dilemmas)	UK	2019	Mixed-method: interview+survey	Various professionals	23; 198	Definition is based on several previous empirical works	No examples of interview questions. In a survey moral distress scale developed by Preshaw et al. (2017)
Piers et al.	6	End-of-life care of the geriatric patient and nurses’ moral distress (moral distress)	Belgium	2012	Quantitative survey	Nurses	222	Adapted from Schwenzer & Wang (2006)	Modified version of Corley’s moral distress scale
Pijl-Zieber et al.	7	Caring in the wake of the rising tide: Moral distress in residential nursing care of people living with dementia (moral distress)	Canada	2018	Quantitative survey	Various professionals	389	Nathaniel definition (2003)	Developed their own moral distress scale based on qualitative interviews
Sedaghati et al.	8	Moral Distress and its Influential Factors in the Nurses of the Nursing Homes in Khorasan Provinces in 2019: A Descriptive-Correlational Study (moral distress)	Iran	2020	Quantitative survey	Nurses	227	Own definition	Developed their own moral distress scale based on earlier measurements
Spenceley et al.	9	Mitigating Moral Distress in Dementia Care: Implications for Leaders in the Residential Care Sector (moral distress)	Canada	2019	Mixed method: interviews+survey	Nurses	389	Nathaniel definition (2003)	The interviews began with a discussion of the definition of moral distress, followed by prompting questions asking participants to recall specific events or times when they experienced moral distress, how they felt during and after the experience, what effects they experienced that they could attribute to moral distress and what helped, or could help, to reduce moral distress. Based on interviews and literature, researchers developed their own moral distress scale.
Spenceley et al.	10	Sources of moral distress for nursing staff providing care to residents with dementia (moral distress)	Canada	2017	Qualitative interviews	Various professionals	18	Jameton’s definition	No information on questions
Wiersma et al.	11	That just breaks my heart’: Moral Concerns of Direct Care Workers Providing Palliative Care in LTC Homes (moral distress)	Ontario Canada	2019	Qualitative focus group interviews	Various professionals	45	Definition is based on previous literature	Open-ended questions: participants were asked their understanding of palliative care and quality of life, the role standing of palliative care and quality of life, the role that they played in providing palliative care, the role that the family played in providing palliative care, how the organizational structure facilitated or hindered their abilities in providing palliative care, and what they would change regarding palliative care delivery in their home if they could.
Villa et al.	12	Moral Distress in Community and Hospital Settings for the Care of Elderly People. A Grounded Theory Qualitative Study (moral distress)	Italy	2021	Qualitative interviews	Various professionals	13	Jameton’s definition	Open-ended questions aimed at understanding how participants deal with a morally distressing event and explore how their actions and choices are limited or enhanced by environmental triggers.
Wocial et al.	13	Factors Associated with Physician Moral Distress Caring for Hospitalized Elderly Patients Needing a Surrogate Decision-maker: a Prospective Study (moral distress)	Pennsylvania, USA	2020	Quantitative survey	Physicians	154	Adapted from Dudinski (2016)	Moral distress thermometer developed by Wocial & Weaver
Young et al.	14	‘Powerlessness’ or ‘doing the right thing’—Moral distress among nursing home staff caring for residents at the end of life: An interpretive descriptive study (moral distress)	England	2017	Qualitative interviews	Various professionals	16	Adapted from Peter (2013)	Interviews started with a reminder of the aim of the research, and the focus was on examining situations where the participants had cared for residents at the end of life, where they had achieved the care outcomes or where they had been unable to achieve them due to circumstances beyond their control.

**Table 4:** Strategies to mitigate moral distress and their categorizations identified in the included studies. Each study is indicated using study numbers in Table 1.

Strategy Main Category	Strategies to Mitigate Moral Distress	Number of Strategies
1. Organizational support and education (in 2 studies)	More resources for care (9), increased education (9), increased administrative/leadership support (9), trust from the leaders (12),	4
2. Peer support (in 2 studies)	Peer support (9), relying on group morality in decision making (12), sharing feelings and choices with colleagues and the team (12)	3
3. Improving selfcare and competence (in 2 studies)	Attention to self-care (9), maintaining soothing behavior (12), understanding how the elderly explicitly or implicitly express their needs (12), psychological support (12)	4
4. Defending patients (in 1 study)	Prioritising respect for autonomy over the principle of beneficence (2), refusing to ask intimate questions (2), defending the interests of the client (2)	3

## Appendix A

**Search strategy in Scopus database**

TITLE-ABS-KEY (aged OR aging OR elder* OR “old* people” OR gerontol* OR geriatr* OR “old age”)

AND

TITLE-ABS-KEY (“elder* care” OR “old-age welfare” OR “care of the elderly” OR “home care services” OR “home care” OR “home help service*” OR “home nursing” OR “assisted living” OR “residential home*” OR “residential care” OR “nursing home*” OR “long term care” OR “Homes for the Aged”)

AND

TITLE-ABS-KEY (personnel OR staff OR work* OR employee* OR professional* OR nurse* OR physician* OR “social worker*”)

AND

TITLE (“moral distress” OR “ethical dilemma*” OR “ethical strain” OR “ethical distress” OR “moral dilemma*” OR “moral strain” OR “moral issue*” OR “ethical issue*” OR “ethical conflict*” OR “moral conflict*” OR “ethical concern” OR “moral concern”)

AND

(LIMIT-TO (DOCTYPE,”ar”)

AND

LIMIT-TO (DOCTYPE,”English”)

**Search strategy in CINAHL database**

“aged” OR “aging” OR “elder*” OR “old people” OR “older people” OR “gerontol*” OR “geriatr*” OR “old age”

AND

“elderly care” OR “eldercare” OR “elder care” OR “old-age welfare” OR “care of the elderly” OR “home care service*” OR “home care” OR “home help service*” OR “home nursing” OR “assisted living” OR “residential home*” OR “residential care” OR “nursing home*” OR “long term care” OR “Homes for the Aged”

AND

“personnel” OR “staff” OR “work*” OR “employee*” OR “professional*” OR “nurse*” OR “physician*” OR “social worker*”

AND

“moral distress” OR “ethical dilemma*” OR “ethical strain” OR “ethical distress” OR “moral dilemma*” OR “moral issue*” OR “ethical issue*” OR “ethical conflict*” OR “moral conflict*” OR “ethical concern” OR “moral concern”

**Search strategy in PUBMED database**

“aged”[Title/Abstract] OR “aging”[Title/Abstract] OR “elder*”[Title/Abstract] OR “old people”[Title/Abstract] OR “older people”[Title/Abstract] OR “gerontol*”[Title/Abstract] OR “geriatr*”[Title/Abstract] OR “old age”[Title/Abstract]

AND

“elderly care”[Title/Abstract] OR “eldercare”[Title/Abstract] OR “elder care”[Title/Abstract] OR “old-age welfare”[Title/Abstract] OR “care of the elderly”[Title/Abstract] OR “home care service*”[Title/Abstract] OR “home care”[Title/Abstract] OR “home help service*”[Title/Abstract] OR “home nursing”[Title/Abstract] OR “assisted living”[Title/Abstract] OR “residential home*”[Title/Abstract] OR “residential care”[Title/Abstract] OR “nursing home*”[Title/Abstract] OR “long term care”[Title/Abstract] OR “Homes for the Aged”[Title/Abstract]

AND

“personnel”[Title/Abstract] OR “staff”[Title/Abstract] OR “work*”[Title/Abstract] OR “employee*”[Title/Abstract] OR “professional*”[Title/Abstract] OR “nurse*”[Title/Abstract] OR “physician*”[Title/Abstract] OR “social worker*”[Title/Abstract]

AND

“moral distress”[Title] OR “ethical dilemma*”[Title] OR “ethical distress”[Title] OR “moral dilemma*”[Title] OR “moral issue*”[Title] OR “ethical issue*”[Title] OR “ethical conflict*”[Title] OR “moral conflict*”[Title] OR “ethical concern”[Title] OR “moral concern”[Title]
